# Transactional sex and HIV risks – evidence from a cross-sectional national survey among young people in Uganda

**DOI:** 10.3402/gha.v8.27249

**Published:** 2015-05-21

**Authors:** Vikas Choudhry, Anne-Emmanuelle Ambresin, Viola Nilah Nyakato, Anette Agardh

**Affiliations:** 1 Social Medicine and Global Health, Department of Clinical Sciences, Lund University, Malmö, Sweden; 2 Multidisciplinary Unit for Adolescent Health, Lausanne University Hospital, Lausanne, Switzerland; 3 Institute of Interdisciplinary Training and Research (IITR), Mbarara University of Science and Technology, Mbarara, Uganda

**Keywords:** Uganda, transactional sex, HIV, sexual coercion, multiple concurrent sexual relationships, young people

## Abstract

**Background:**

Transactional sex is associated with the HIV epidemic among young people in Uganda. Few quantitative studies based on nationally representative survey data explored the relationship between sexual behaviors, HIV infection, and transactional sex.

**Objective:**

This study aimed to determine the associations between risky sexual behaviors, participation in transactional sex, and HIV sero-status among men and women aged 15–24 in Uganda.

**Design:**

The study uses data from the Uganda AIDS Indicator Survey, a cross-sectional national HIV serological study conducted in 2011. We analyzed data on 1,516 men and 2,824 women aged 15–24 who had been sexually active in the 12 months preceding the survey. Private, face-to-face interviews were also conducted to record the sociodemographics, sexual history, and experiences of sexual coercion. Logistic regression analysis was performed to measure associations between sexual behaviors and transactional sex, and associations between HIV sero-status and transactional sex.

**Results:**

Among young people who had been sexually active in the 12 months prior to the survey, 5.2% of young men reported paying for sex while 3.7% of young women reported receiving gifts, favors, or money for sex. Lower educational attainment (OR_adjusted_ 3.25, CI 1.10–9.60) and experience of sexual coercion (OR_adjusted_ 2.83, CI 1.07–7.47) were significantly associated with paying for sex among men. Multiple concurrent sexual relationships were significantly associated with paying for sex among young men (OR_adjusted_ 5.60, CI 2.08–14.95) and receiving something for sex among young women (OR_adjusted_ 8.04, CI 2.55–25.37). Paying for sex among young men and having three to five lifetime sexual partners among young women were associated with increased odds of testing positive for HIV.

**Conclusions:**

Transactional sex is associated with sexual coercion and HIV risk behaviors such as multiple concurrent sexual partnerships among young people in Uganda. In addition, transactional sex appears to place young men at increased risk for HIV in Uganda. Both sexes appear equally vulnerable to risks associated with transactional sex, and therefore should be targeted in intervention programs. In addition, strengthening universal education policy and improving school retention programs may be beneficial in reducing risky sexual behaviors and transactional sex.

Although HIV transmission is declining worldwide, in young people (aged 15–24) it remains a growing concern. In 2013, 33% of all new HIV infections, globally, occurred in people aged 15–24, and young women were disproportionately more affected than young men (1, 2). The risk of HIV transmission is increased by the social and the developmental context surrounding young people. These factors include educational and vocational opportunities, economic disparities, gender inequality, partnership formation, power dynamics within relationships, time and characteristics of sexual initiation, and biological factors such as being of the female sex and pubertal timing (3, 4). Transactional sex and age-disparate sexual relationships seem to be additional drivers of HIV in the sub-Saharan region of Africa, where Uganda is located (5–7). The increase in HIV prevalence among young people in Uganda, from 3% in 2004–2005 to 4% in 2011, indicates that HIV incidence might be increasing in this age group. The prevalence of HIV in Uganda has a significant gender difference with HIV prevalence among women aged 15–24 years being 5%, while it is 2% among men in the same age group (8).

Transactional sex is defined as the exchange of favors, gifts, or money for sexual activity (9, 10). In public health, transactional sex is often differentiated from commercial sex work since participants do not identify themselves as ‘prostitutes’ and ‘clients’. Exchanging gifts for sex is often a part of a broader set of obligations that might not involve a predetermined payment or contract (11). The meaning attached to transactional sex varies widely within sub-Saharan Africa. The exchange of gifts or money for sex may signify: 1) a committed relationship, 2) an acknowledgement of respect, 3) an expression of affection, 4) an obligation fulfilled, or 5) maybe a display to impress other men (9, 12–14). The motivations for transactional sex exist along a continuum of needs from financial vulnerabilities (‘survival sex’) on one end to material desires, including higher social status, jewelry, mobile phones, and so on (‘consumption sex’) on the other (14).

Transactional sex is commonly described as a partnership between a younger woman and an older man (or ‘sugar daddy’). It is characterized by a power differential in favor of the man (9, 10), although ‘sugar daddies’ are not as widespread as often assumed (5). Transactional sex has also been reported among youths close in age where it may be characterized as a casual partnership. The exchange of gifts and money has been described as an incentive in regular partnerships, too (5, 6, 12, 15, 16).

Transactional sex, especially among young women, has been linked to poor sexual and reproductive health outcomes as unintended pregnancies, unsafe abortions, sexually transmitted infections (including HIV), and sexual coercion (7, 17–19). Transactional sex among young men and older women (or sugar mommies) are also characterized by economic and power asymmetries and are often associated with increased HIV risk behaviors such as inconsistent condom use and multiple concurrent sexual partners of varying ages (20, 21). Research conducted among men supports the association between sexual coercion and transactional sex but these studies have been few and limited to sub-populations of men in universities, schools, or drinking establishments (12, 22).

Despite the growing literature in Uganda on transactional sex, most investigations have been qualitative and are aimed at understanding 1) the motivations of young women who participate in such behaviors and 2) existing economic and power asymmetries within these relationships (23, 24). In addition, although transactional sex is prevalent among young people in Uganda, the restricted scope of the majority of these studies raises the question of generalizability (20, 25, 26). Given the increasing prevalence and incidence of HIV from 2004 to 2011 among young people in Uganda, along with the growing trend of transactional sex among young people, a better understanding of the sexual behaviors in this sub-group is needed to develop effective interventions.

The aim of the study is three-fold: first to examine the prevalence of receiving gifts, favors, or money for sex among young women aged 15–24 and paying for sex among young men aged 15–24 in Uganda; second, to determine the associations between sexual risk behaviors and both forms of transactional sex; and third, the correlation between participation in either form of transactional sexual behavior and HIV sero-status among this population.

## Methods

### Study design and setting

Data for this analysis were drawn from the Uganda AIDS Indicator Survey (UAIS), a nationally representative, population-based survey of HIV serological status. It was carried out between February and September 2011 by the Ministry of Health (MoH), Uganda, in collaboration with ICF International, US, and the Uganda Bureau of Statistics (UBOS) (8).

A representative probability sample of 11,750 households was selected for the survey, which used a two-stage sample design. The first stage involved selecting clusters from the list of enumeration areas covered in the 2002 population census. This resulted in identification of 470 clusters consisting of 79 urban and 391 rural points. The second stage involved systematic sampling of 25 households per cluster from the list of households produced by UBOS.

All women and men aged 15–59, who were either permanent residents of a household or visited the household on the night before the survey, were eligible for inclusion in the interviews. In total, 12,153 women (response rate 98.0%) and 9,588 men (response rate 96.0%) were interviewed. Informed consent was obtained prior to conducting the interviews and obtaining blood samples for HIV and syphilis testing.

The contents of the questionnaires were based on 1) the standardized AIDS Indicator Survey (AIS) questionnaire developed by Demographic Health Survey (DHS) and 2) the questionnaire used in 2004–2005 Uganda HIV/AIDS Sero-Behavioral Survey (8). Data collection included sociodemographic characteristics (age, marital status, area of residence, highest educational level, religion, economic level of the household, and employment status), self-reported sexual behaviors, and sexual coercion. Questions on transactional sex were based on the sex of the individual. Age of recent sexual partner and whether gifts, favors, or money were received in exchange for sex were asked to women only; the questions about having paid in exchange for sex were asked to men only.

All respondents who were interviewed were asked to voluntarily provide blood samples for testing for HIV and syphilis. Of all individuals eligible for interview and testing, 96.8% of the women and 94.1% of the men could be tested. Home based rapid HIV testing was done based on national protocols. To obtain informed consent for blood collection, the field team explained the procedure, the confidentiality of the data, the fact that respondents could obtain their test results immediately if they wished, that they would be provided with counseling before and after the rapid tests, and that, if they tested positive for HIV, they could obtain their CD4 count from a nearby health facility. Finally, if respondents had any further questions or wanted to lodge a complaint, a card with contact information of the three principal investigators of the survey and the chair of the ethics committee was provided to all respondents. For non-emancipated respondents aged 15–17 (i.e. those who still live with other adults), laboratory technicians also sought consent of the parent or guardian in addition to the respondent.

The sample used in this study was acquired through the merging and appending of four data sets downloaded from the MEASURE DHS website. These were the standard individual data set containing sociodemographic information for males and females, a household member data set with information on all household members and characteristics, and AIS data set that had HIV test results along with other indicators for HIV monitoring. Data from individual interviews and HIV sero-status were linked through unique identifiers for each respondent in the data set. A final data set, which included respondents who were aged between 15 and 24 and HIV results, was used in the final analysis. Further details on the survey content, methodology, HIV testing, and data collection and processing have been published elsewhere (8).

The protocol for blood specimen collection and analysis was reviewed and approved by the Science and Ethics Committee of the Uganda Virus Research Institute, ICF Macro's Institutional Review Board, and a review committee at the US Centers for Disease Control and Prevention (CDC) in Atlanta, Georgia. The Uganda National Council of Science and Technology ethics committee also approved the national survey. Permission to use the data for this study was obtained from ICF International.

### Study measures

#### Outcome variables

*Transactional sex among young women* was based on the following questions: 1) ‘Did you ever give sex in exchange for goods or services? 2) Did you ever give sex in exchange for money? 3) Did this happen in the last 12 months?’ The above questions were limited to women in the sample. If the respondent answered ‘yes’ for either question 1 or 2 and ‘yes’ to question no. 3, she was categorized as Having received money or gifts in return for sex in the last 12 months and otherwise categorized as Not having received money or gifts in return for sex in the last 12 months.

*Transactional sex among young men* was based on the question: ‘In the last 12 months, did you pay anyone in exchange for having sexual intercourse?’ The question was limited to men in the sample and the options were categorized as Having paid for sex in last 12 months or Not having paid for sex in last 12 months.

*HIV sero-status* was categorized as ‘HIV positive’ or ‘HIV negative’, depending on test results from the blood samples of individual respondents. One case with an indeterminate HIV test result was not considered for analysis.

#### Sexual behaviors variables

*Lifetime number of sexual partners* was based on the question: ‘In total, with how many different people have you had sexual intercourse in your lifetime?’ The options were then categorized into ‘≤2 partners’, ‘3–5 partners’, or ‘>5 partners’.

*Multiple and concurrent sexual partnerships in the last 12 months* were defined as ‘<2 partners’, ‘≥2 partners but not concurrent’, or ‘≥2 partners and concurrent’. The 2011 UAIS collected information on the time since the first and most recent sexual intercourse with each sexual partner in the 12 months before the survey. This information was used to determine if sexual intercourse with one partner occurred between two acts of intercourse with another partner, that is, whether two partnerships are concurrent. Cumulative concurrent partnerships were defined as participation in overlapping sexual partnerships in the 12 months before the UAIS survey was conducted.

*Age at sexual debut* was based on the question: ‘How old were you when you had sexual intercourse for the first time?’ The options were divided into two categories ‘<15 years’ and ‘≥15 years’, based on the WHO definition of risky sexual debut.

*Condom use at last sexual activity with most recent partner* was categorized as ‘yes’ or ‘no’.

*Condom use at last sexual activity with the transactional partner* was categorized as ‘yes’ or ‘no’. This question was limited to young people that responded ‘yes’ for having paid or received money or gifts in exchange for sex.

*Ever experienced sexual coercion* was based on the response ‘yes’ to any of the following questions: ‘Were you ever physically forced to have sex against your will?’ or ‘Were you ever coerced to have sex, that is, against your will but without the use of physical force?’ For ethical reasons, the questions on sexual coercion and violence were restricted to one randomly selected male or female member per household. In our study, 1,010 women and 522 men were included in the analysis for this variable.

*Age of recent partner* was categorized as ‘younger or same age’, ‘older by 5–9 years’, or ‘≥10 years older’.

#### Sociodemographic variables

*Age* was categorized as ‘15–19 years’ and ‘20–24 years’.

*Area of residence* was recorded as ‘urban’ or ‘rural’ based on the respondent's current place of residence.

*Educational attainment of the respondent* was categorized as ‘>primary’ or ‘≤primary’.

*Marital status* was classified as ‘never married’, ‘currently married or living with partner’, or ‘divorced, widow (ed), or separated’.

*Employment status of the respondent in the past 12 months* was based on the question ‘Have you done any work in the last 12 months?’ and was classified as ‘yes’ or ‘no’.

*Household wealth index* in the original data set was based on dividing the households into five different quintiles based on household assets, using principal component analysis. The divided wealth quintiles were then coded as a 5-point ordinal scale with the higher points indicating higher household wealth (8). The households in the fourth and the highest quintiles were clubbed together as ‘belonging to the richest wealth quintile’, the middle category as ‘belonging to the middle wealth quintile’, and households in lowest and the second wealth quintiles as ‘belonging to the poorest wealth quintiles ’.

*Religious affiliations* were categorized as ‘Catholic’, ‘Protestant’, ‘Other Christians, including Pentecostals and Seventh Day Adventists’, ‘Muslims’, and ‘Other religions’.

### Statistical analysis

The statistical analysis was done using IBM-SPSS Version 20.0. Confidence intervals (CI) were calculated at the 95% level to indicate statistical significance. Analysis was restricted to men and women aged 15–24 who had been sexually active in the past 12 months. Descriptive analyses were carried out and summarized by gender and by respondent's self-reporting of participation in transactional sex (receiving gifts, favors, or money for sex in the case of women and paying for sex among men). The percentages reported in the descriptive tables were weighted in order to account for the complex survey methodology (8).

Bivariate logistic regression analysis was performed to calculate the unadjusted odds ratio (OR_crude_) to determine associations between risky sexual behaviors and the self-reported receipt of gifts, favors, or money for sex among women, and paying for sex among men.

A step-wise multivariate logistic regression analysis was performed to calculate the adjusted odds ratio (OR_adjusted_) to determine the associations between risky sexual behaviors and self-reported transactional sex among men and women. The selection of variables for multivariate logistic regression was based on a review of literature and on those variables that were found in bivariate analysis to be statistically significantly associated with reporting receiving gifts, favors, or money and paying in exchange for sex. All factors were included in the final model to facilitate comparisons across the reporting of either form of transactional sex. Colinearity of risky sexual behaviors was also assessed, as a result of which the lifetime number of sexual partners was removed from the final model. The sociodemographic characteristics included in the analyses were age, marital status, area of residence, and educational attainment of the respondent.

Finally, logistic regression analysis was performed to estimate the association between HIV-positive sero-status and reporting risky sexual behaviors, including transactional sex, before and after adjustment for sociodemographic factors and sexual behaviors such as lifetime number of sexual partners and age at sexual debut. Age of recent partner was an additional variable that was adjusted for in the multivariate logistic regression analysis for HIV sero-status among women.

## Results

A total of 3,479 men and 4,627 women aged between 15 and 24 across Uganda were selected. We included only those individuals who had been sexually active in the 12 months before the survey (43.6% men, 61.5% women). HIV prevalence in our study was found to be 2.7% among young men and 6.2% among young women (data not shown).

Table 1 presents the distribution of sociodemographic characteristics among the sample of young people in Uganda who had been sexually active in the 12 months prior to the survey by gender and paying for sex (men only) or receiving gifts, favors, or money for sex (women only). In the study sample, 5.2% of the men reported paying for sex, while 3.7% of the women reported having received gifts, favors, or money in exchange for sex. A higher percentage of young men who reported paying for sex belonged to the age group 20–24, came from rural area, had never been married, and had a lower educational attainment. There was a greater prevalence of paying for sex among working-class males and those who belonged to the middle two wealth quintiles. An almost equal percentage of women aged between 15–19 and 20–24 reported receiving gifts, favors, or money in exchange for sex. The majority of those young women, who reported receiving gifts, favors, or money in exchange for sex were employed, came from rural residence, and had a lower educational attainment.

**Table 1:** Sociodemographic characteristics of sexually active young people in Uganda 12 months prior to the survey

	Men 15–24 (*N*=1,516)		Women 15–24 (*N*=2,824)	
		
Characteristics	Did not pay for sex (*N*=1,437) *n* (%)	Paid for sex (*N*=79) *n* (%)	Received no gifts, favors, or money for sex (*N*=2,719) *n* (%)	Received gifts, favors, or money for sex (*N*=105) *n* (%)
Age groups				
15–19	459 (31.9)	24 (30.8)	915 (33.7)	54 (51.4)
20–24	978 (68.1)	54 (69.2)	1,804 (66.3)	51 (48.6)
Area of residence				
Urban	315 (21.9)	17 (21.5)	641 (23.6)	22 (21.0)
Rural	1,122 (78.1)	62 (78.5)	2,077 (76.4)	83 (79.0)
Educational attainment				
>Primary school	598 (41.6)	26 (32.9)	927 (34.1)	20 (19.0)
≤Primary school	839 (58.4)	53 (67.1)	1,791 (65.9)	85 (81.0)
Marital status				
Never married	873 (60.8)	48 (60.7)	681 (25.0)	46 (43.8)
Married	501 (34.9)	24 (30.4)	1,830 (67.3)	42 (40.0)
Divorced/widowed/separated	63 (4.3)	7 (8.9)	208 (7.7)	17 (16.2)
Employment status in past year				
Working	1,177 (81.9)	68 (86.1)	1,785 (65.6)	65 (62.5)
Not working	260 (18.1)	11 (13.9)	934 (34.4)	39 (37.5)
Household wealth index				
Richest two quintiles	642 (44.7)	38 (48.3)	1,292 (47.5)	49 (46.7)
Middle quintile	265 (18.4)	26 (32.9)	459 (16.9)	21 (20.0)
Poorest two quintiles	530 (36.9)	15 (18.8)	968 (35.6)	35 (33.3)
Religious affiliations				
Catholic	590 (41.1)	29 (37.2)	1,055 (38.8)	45 (42.9)
Protestant	477 (33.2)	33 (42.3)	918 (33.8)	41 (39.0)
Other Christians	131 (9.1)	7 (9.0)	334 (12.3)	8 (7.6)
Muslim	226 (15.7)	9 (11.5)	394 (14.5)	10 (9.5)
Other religion	12 (0.9)	0 (0.0)	17 (0.6)	1 (1.0)

(All percentages are weighted).

Table 2 shows sexual behaviors and HIV status among young people in Uganda by participation in transactional sex. A greater percentage of those men who reported paying for sex, as compared to women who reported having received gifts, favors, or money for sex, had more than five lifetime sexual partners (males 65.8% vs. females 12.4%), concurrency of sexual partners in the past 12 months (44.9% vs. 15.2%), and did not use a condom at the last sexual activity (34.6% vs. 21.9%). Among those individuals who reported transactional sex, a greater percentage of women, as compared to men, also reported being sexually coerced (females 36.4% vs. males 28.6%) and did not use a condom with their transactional partner at their last sexual activity (49.0% vs. 32.0%).

**Table 2:** Sexual behaviors and HIV status of sexually active young people in Uganda 12 months prior to the survey

	Men 15–24 (*N*=1,516)		Women 15–24 (*N*=2,824)	
		
Characteristics	Did not pay for sex (*N*=1,437) *n* (%)	Paid for sex (*N*=79) *n* (%)	Received no gifts, favors, or money for sex (*N*=2,719) *n* (%)	Received gifts, favors or money for sex (*N*=105) *n* (%)
Life time number of sexual partners				
≤2 partners	595 (41.4)	8 (10.1)	2,047 (75.3)	52 (49.5)
3–5 partners	549 (38.2)	19 (24.1)	624 (22.9)	40 (38.1)
>5 partners	294 (20.4)	52 (65.8)	48 (1.8)	13 (12.4)
Multiple and concurrent sexual partnerships in the past 12 months				
<2 partners	1,143 (79.6)	28 (35.9)	2,605 (95.8)	76 (72.4)
≥2 partners but not concurrent	110 (7.7)	15 (19.2)	35 (1.3)	13 (12.4)
≥2 partners and concurrent	183 (12.7)	35 (44.9)	79 (2.9)	16 (15.2)
Age at sexual debut				
≥15	1,194 (83.1)	51 (64.6)	2,231 (82.1)	68 (64.8)
<15	243 (16.9)	28 (35.4)	488 (17.9)	37 (35.2)
Condom use at the last sexual activity with the recent partner				
Yes	966 (67.2)	51 (65.4)	2,306 (84.8)	82 (78.1)
No	471 (32.8)	27 (34.6)	413 (15.2)	23 (21.9)
Sexual coercion^a^				
No	442 (89.7)	20 (71.4)	751 (77.6)	21 (63.6)
Yes	51 (10.3)	8 (28.6)	217 (22.4)	12 (36.4)
HIV status				
Negative	1,375 (98.0)	66 (83.5)	2,490 (94.0)	92 (88.5)
Positive	28 (2.0)	12 (15.2)	160 (6.0)	12 (11.5)
Condom use at the last sexual activity with transactional partner				
Yes	–	41 (68.0)	–	53 (51.0)
No	–	20 (32.0)	–	52 (49.0)

All percentages are weighted.

a Data include respondents questioned about sexual coercion (*n*=522 men and *n*=1,010 women).

Table 3 presents the bivariate logistic regression analysis between sexual behaviors associated with reporting paying for sex among men and reporting receiving gifts, favors, or money in exchange for sex among women. Young men and young women who reported more than five lifetime partners were 14 times and 11 times, respectively, more likely to report having participated in transactional sex compared with their counterparts with less than two lifetime sexual partners. Similarly, young men and young women who reported concurrency of two or more sexual partners in the past 12 months were almost seven and eight times, respectively, at higher odds of reporting transactional sex in the 12 months prior to the survey. Sexual coercion was associated with almost five times the risk for reporting transactional sex among young men. Young women who had experienced sexual coercion carried almost twice the odds for reporting transactional sex, although this association was not statistically significant.

**Table 3:** Bivariate association (Crude Odds Ratio [OR_crude_]; 95% Confidence Interval [CI]) between risky sexual behaviors and reporting transactional sex among sexually active young people in Uganda 12 months prior to the survey

	Men 15–24 Paid for sex in the past 12 months		Women 15–24 Received gifts, favors, or money for sex in the past 12 months	
		
Characteristics	OR_crude_	95% CI	OR_crude_	95% CI
Life time sexual partners				
≤2 partners	1 (Ref)		1 (Ref)	
3–5 partners	**2.73**	**1.16–6.40**	**2.53**	**1.66–3.86**
>5 partners	**14.00**	**6.44–30.45**	**10.80**	**5.50–21.14**
Multiple and concurrent sexual partnerships in the past 12 months				
<2 partners	1 (Ref)		1 (Ref)	
≥2 partners but not concurrent	**5.60**	**2.92–10.80**	**13.38**	**6.85–26.11**
≥2 partners and concurrent	**7.80**	**4.64–13.12**	**6.78**	**3.75–12.24**
Age at sexual debut				
≥15 years	1 (Ref)		1 (Ref)	
<15 years	**2.40**	**1.43–4.05**	**2.44**	**1.62–3.70**
Condom use at the last sexual activity with the recent partner				
Yes	1 (Ref)		1 (Ref)	
No	1.13	0.70–1.90	1.55	0.97–2.50
Sexual coercion^a^				
No	1 (Ref)		1 (Ref)	
Yes	**4.73**	**2.00–11.13**	1.93	0.94–4.00

a Data include respondents questioned about sexual coercion (*n*=522 men and *n*=1,010 women).

Figures in bold are significant at 5% level.

Table 4 reports the multivariate logistic regression analysis between various factors associated with transactional sex in men and women. Lower educational attainment was associated with paying for sex among young men (OR_adjusted_ 3.25, CI 1.10–9.60). Young men who had experienced sexual coercion had an almost three times greater OR of paying for sex in the past 12 months (OR_adjusted_ 2.83, CI 1.07–7.47) than men who had not experienced sexual coercion. Having had more than two sexual partners with concurrency in the past 12 months was associated with having paid for sex among young men (OR_adjusted_ 5.60, CI 2.08–14.95) and with having received gifts, favors, or money in exchange for sex among young women (OR_adjusted_ 8.04, CI 2.55–25.37).

**Table 4:** Multivariate logistic regression analysis (Adjusted Odds Ratio [OR_adjusted_]^a^; 95% Confidence Intervals [CI]) for reporting transactional sex among sexually active young people in Uganda 12 months prior to the survey

	Men 15–24 Paid for sex in the past 12 months		Women 15–24 Received gifts, favors, or money for sex in the past 12 months	
		
Characteristics	OR_adjusted_	95% CI	OR_adjusted_	95% CI
Age groups				
15–19	1 (Ref)		1 (Ref)	
20–24	1.60	0.50–5.02	1.50	0.65–3.36
Area of residence				
Urban	1 (Ref)		1 (Ref)	
Rural	0.83	0.25–2.70	1.25	0.46–3.44
Educational attainment				
>Primary school	1 (Ref)		1 (Ref)	
≤Primary school	**3.25**	**1.10–9.60**	1.73	0.65–4.60
Marital status				
Never married	1 (Ref)		1 (Ref)	
Married	0.40	0.13–1.20	0.60	0.22–1.60
Divorced/widowed/separated	1.25	0.30–5.80	1.50	0.40–6.00
Multiple and concurrent sexual partnerships in the last 12 months				
<2 partners	1 (Ref)		1 (Ref)	
≥2 partners but not concurrent	**5.32**	**1.70–16.04**	**8.90**	**2.50–31.20**
≥2 partners and concurrent	**5.60**	**2.08–14.95**	**8.04**	**2.55–25.37**
Age at sexual debut				
≥15 years	1 (Ref)		1 (Ref)	
<15 years	0.50	0.20–1.59	1.34	0.60–3.20
Condom use at the last sexual activity				
Yes	1 (Ref)		1 (Ref)	
No	0.84	0.32–2.23	2.15	0.80–5.95
Sexual coercion^b^				
No	1 (Ref)		1 (Ref)	
Yes	**2.83**	**1.07–7.47**	1.86	0.85–4.08

a All variables mutually adjusted.

b Data include respondents questioned about sexual coercion (*n*=522 men and *n*=1,010 women).

Figures in bold are significant at 5% level.

Table 5 reports associations between sexual behaviors (including transactional sex) and HIV status among young men and women. Paying for sex was significantly associated with HIV-positive sero-status among men, even after adjusting for sociodemographic and other risky sexual behaviors (OR_adjusted_ 8.30, CI 3.64–18.86). Among women, having three to five lifetime sexual partners (OR_adjusted_ 2.12, CI 1.50–3.03) was associated with being HIV positive after adjusting for sociodemographic and other risky sexual behaviors.

**Table 5:** Unadjusted odds ratio (OR_crude_) and adjusted odds ratio (OR_adjusted_) at 95% confidence interval (CI) for HIV-positive sero-status among sexually active young people in Uganda 12 months prior to the survey

	Men 15–24		Women 15–24	
		
Characteristics	OR_crude_ (95% CI)	OR_adjusted_ (CI)^a^	OR_crude_ (95% CI)	OR_adjusted_ (95% CI)^a^
Transactional sex in the last 12 months^b^				
No	1 (Ref)	1 (Ref)	1 (Ref)	1 (Ref)
Yes	**8.72 (4.30–18.00)**	**8.30 (3.65–18.86)**	**1.97 (1.05–3.70)**	1.65 (0.82–3.33)
Lifetime number of sexual partners	1 (Ref)	1 (Ref)	1 (Ref)	1 (Ref)
≤2				
3–5	2.09 (0.90–4.93)	1.46 (0.62–3.44)	**2.57 (1.87–3.53)**	**2.12 (1.50–3.03)**
>5	**3.40 (1.42–8.08)**	1.60 (0.62–4.08)	**2.60 (1.13–6.00)**	2.24 (0.99–5.01)
Age of sexual debut				
≥15	1 (Ref)	1 (Ref)	1 (Ref)	1 (Ref)
<15	0.82 (0.35–2.00)	0.62 (0.24–1.60)	1.20 (0.85–1.80)	1.14 (0.76–1.70)
Age of recent partner^c^	–	–		
Younger or same age			1 (Ref)	1 (Ref)
Older by 5–9 years			1.06 (0.74–1.53)	1.04 (0.72–1.50)
≥10 years older			**1.78 (1.22–2.60)**	1.50 (0.99–2.30)

a All variables mutually adjusted for each other and for age, area of residence, educational attainment, and marital status.

b Transactional sex in men was defined as paying for sex and in women was defined as receiving gifts, favors, or money for sex.

c Data unavailable for male respondents.

Figures in bold are significant at 5% level.

## Discussion

Despite decades of HIV prevention efforts targeting transactional sex, we still found the prevalence of transactional sex among young men and women to be considerable. Young men had a higher prevalence of paying for sex compared to women who received gifts, favors, or money in exchange for sex. Lower educational attainment and having experienced sexual coercion was associated with paying for sex among young men. Multiple concurrent sexual partnerships were associated with transactional sex for men and women. Paying for sex was associated with HIV-positive sero-status among young men while a higher number of lifetime sexual partners was associated with HIV-positive sero-status among young women.

A study done in 12 sub-Saharan African countries reported that in-school status was not associated with participation in transactional sex among young men (9). However, a study analyzing associations between community environments and risky transactional sex, among sexually active men in Malawi, Nigeria, and Tanzania, reported less likelihood of participation in risky transactional sex with increasing educational levels. Low level of education among men can possibly be a barrier to receive information on safe sexual behaviors in transactional sexual relationships (25). Our findings are in line with the literature suggesting the lack of education as a risk factor for risky sexual behaviors (26).

We also found the experience of sexual coercion to be associated with paying for sex among young men. This corresponds with the results of another study of male students in Uganda (22). Experience of sexual coercion or violence, particularly during childhood, has also been associated with the perpetration of rape in adulthood (27, 28). Furthermore, unwanted sexual experiences may reduce the ability to initiate and sustain emotionally stable relationships with the opposite sex and may encourage a preference for informal, impersonal sexual interaction as occurs in transactional sex (29).

Men may also engage in transactional sex with other men. Those individuals represent a substantially different population than those who engage in transactional sex with women. A study in South Africa indicated that among males, an experience of sexual coercion from a member of the same sex was strongly associated with paying for sexual services (30). In countries such as Uganda, where homosexuality is stigmatized, access to healthcare and HIV prevention services may be limited for men who engage in transactional sex with other men, thereby increasing their vulnerability to HIV as well as increasing the spread of the disease (31). Unfortunately, most studies including our own, fail to identify particular experiences and contexts of sexual coercion and transactional sex among men who have sex with men. Qualitative studies may be able to define the complex relationship between sexual coercion, transactional sex, and HIV risk in young men.

The results of our study indicate that paying for sex among men is associated with multiple concurrent sexual relationships that have been considered as important driver of HIV epidemic in sub-Saharan Africa (32, 33). The sociocultural constructions of masculinity among youth in sub-Saharan Africa, including Uganda, are often deeply rooted in social scripts of achieving manhood through assuming a provider role in sexual relationships and having multiple female sexual partners (12, 34). Paying for sex may be understood in the broader context of this idea of masculinity (27, 30).

We also found that multiple concurrent sexual partnerships were associated with receiving gifts, favors, or money for sex among young women. Anthropological studies conducted in southern Africa often demonstrate the sociocultural scripts of gender roles in which men are providers and women are receivers. Such roles are primary drivers of multiple and concurrent sexual partnerships (35–37). Thus, young women may enter various sexual partnerships, often concurrently, to fulfill multiple needs and desires (11, 14). Moreover, qualitative and quantitative studies, across sub-Saharan Africa, have demonstrated multiple concurrent heterosexual partnerships and little or no condom use within transactional sexual partnerships (5, 7, 13, 14, 18, 24, 26). In a meta-analytical review of studies, exposure to condom promotion messages in HIV prevention strategies was found to have great potential to reduce partners among those trading in sex, especially when delivered as part of a comprehensive risk reduction program (38). A study among Ugandan adolescents also indicated that condom promotion messages in early adolescence results in establishing a safe and lasting behavior pattern in young people (39). Hence, there is a need to emphasize the development of comprehensive risk reduction strategies that not only include components aimed at reducing multiple and concurrent sexual partners but also include negotiation and communication skills aimed at consistent condom use among young people.

Transactional sex has been identified as one of the risk factors for increased vulnerability to HIV. We estimate HIV prevalence among young people reporting sexual activity and transactional sex in the past 12 months from the survey (2.7% men, 6.2% women) to be much higher than the national prevalence of HIV in this age group (1.2% men, 4.0% women). Our findings also reveal that young men who pay for sex are vulnerable to HIV infection. Interventions for transactional sex must, therefore, also include young men and disseminate information on safe sexual practices and the risks associated with transactional sexual relationships. However, our study could not demonstrate an independent association between transactional sex and HIV-positive sero-status among young women after adjustment for risky sexual behaviors, although our findings on HIV prevalence were contrary to a South African study that reported the independent association of transactional sex with HIV incidence after adjustment for number and age of partners (40). We believe that the structural factors, such as economic inequalities in women, along with control of resources by men, patriarchal society, and social norms around acceptability of transactions in return for sex, and risky sexual behaviors associated with transactional sex make young women especially vulnerable to HIV (41–43). Major efforts should be placed on interventions that target young men and women and use a gender transformative approach (GTA). GTA can help challenge implicit assumptions of gender roles and social norms that surround and encourage transactional sex, multiple sexual relationships, and inconsistent condom use (44).

In a longitudinal study done on HIV incidence among young people in Rakai in Uganda, being a student significantly decreased the risk for of HIV infection in men and women (42). Our findings seem to suggest focusing on decreasing school drop out rates and enhancing school attendance as a method of reducing HIV risk behaviors in youth is justified. Interventions such as conditional cash transfers that can mediate the economic drivers of sexual exchange and increase school retention, particularly among young girls, may play an important role in decreasing HIV infections (45).

### Limitations

There are several limitations to our study. Due to the cross-sectional nature of its design, the analyses cannot determine causality but only associations.

Our categorization of whether respondents gave or received the gifts, favors, or money in exchange for sex is based on gender of the respondent. A previous study done at a university in Uganda has shown that both forms of transactional sex, that is, giving or receiving something in exchange for sex, are prevalent among men and women in Uganda (22). Hence, we believe that questions pertaining to transactional sex should be similar for men and women to develop parallel measures and indicators for both sexes. In addition, since the questions about participating in transactional sex asked the male respondent if they had paid for sex, it may have placed the focus on formal monetary exchange, such as in commercial sex work and omitted transactional sex within relationships. Recently, STRIVE, a research program consortium at the London School of Hygiene and Tropical Medicine that is working on structural drivers and pathways to HIV, recommended a set of additional questions pertaining to transactional sex for DHS surveys across sub-Saharan Africa (46).

The data used in our study were obtained by retrospective self-reporting and may have been influenced by recall bias. Sensitive and socially devalued behaviors such as transactional sex, risky sexual behaviors, and experiences of sexual coercion may have been under-reported in face-to-face interviews, but it would be difficult to ascertain if such under-reporting was differential and, therefore, impossible to guess the effect on the findings of the study.

## Conclusions

Transactional sex is associated with HIV risk behaviors such as multiple concurrent sexual partners and sexual coercion among young people in Uganda. Multiple and concurrent sexual partnerships, and exchanging sex for gifts, favors, or money create a fertile ground for HIV transmission. Transactional sex appears to be an important driver of the HIV epidemic, especially among young men. Research, policy, and programmatic interventions should address the intertwined role of sexual coercion, educational attainment, and the risks of engaging in transactional sex among young women and men. In interventions aimed at reducing transactional sexual relationships and sexual coercion, young men should be a part of intervention programs that traditionally have focused on young women only. Universal primary education policy and increased school retention appears to be a good strategy to enhance safe sexual behaviors, reduce transactional sexual relationships, and HIV prevention. Interventions to reduce HIV transmission should incorporate comprehensive risk reduction strategies aimed at decreasing behavioral risks for HIV transmission including reducing multiple concurrent partnerships and promoting consistent condom use. Such interventions should take into account structural inequalities and involve young people to challenge traditional and cultural norms that underlie risky sexual interactions among young people in Uganda.
